# Research on the Matching Relationship between the Supply of Urban Ecological Recreational Space and the Demand of Residents—A Case Study of an Urban Development Area in Wuhan

**DOI:** 10.3390/ijerph19020816

**Published:** 2022-01-12

**Authors:** Xin Xu, Jing Hu, Li Lv, Jiaojiao Yin, Xiaobo Tian

**Affiliations:** 1 The College of Urban and Environmental Science, Central China Normal University, Wuhan 430079, China; xxingeo@163.com (X.X.); lilv@mails.ccnu.edu.cn (L.L.); Jiaojiao.Yin@colostate.edu (J.Y.); txbct3@126.com (X.T.); 2 Wuhan Branch of China Tourism Academy, Wuhan 430079, China

**Keywords:** UERS, spatial justice, supply–demand balance, urban development area of Wuhan

## Abstract

An urban ecological recreational space (UERS), which connects the natural environment with urban residents, is an important guarantee for developing a livable city and improving the well-being of residents. However, there is a serious imbalance between the supply of UERSs and the demand of residents in many big, rapidly developing cities. Previous studies usually used indicators such as scale or quantity to measure the supply level of UERS enjoyed by residents, ignoring its own quality differences. Therefore, taking the urban development area of Wuhan as the research object, we measured the quality of UERS from four dimensions using the entropy method and designed a method to measure the supply service level under the hierarchical travel threshold to analyze the supply level of UERSs based on a community unit. Finally, combined with the demand characteristics of different groups, the matching relationship between supply and demand of UERSs in each community is quantitatively analyzed. The results show the following: (1) The quality of UERS in urban development area of Wuhan varies greatly and its distribution is extremely uneven. (2) The level of supply services and the demand level vary greatly, and the overall performance has a trend of decreasing from the city center to the periphery. (3) The overall matching relationship between supply and demand of UERS is not ideal, and more than half of the communities are in supply deficit or without services. Our study provides a novel perspective on quantifying the supply–demand relationship of UERS. It can more accurately guide decision-makers and planners in determining areas with mismatches between the supply and demand of UERSs and in making targeted layouts of UERSs and relevant policies.

## 1. Introduction

The purpose of urban spatial justice is to guarantee the equality of spatial rights and benefits of people; to meet the basic needs of spaces enjoyed by different groups; and to realize justice in urban space production, space exchange, and space resource allocation [1,2]. Urban ecological recreational space (UERS), as the most important urban ecological infrastructure, is not only an important part of urban spatial structures but also an important standard to measure the construction of urban ecological civilization and the quality of residents’ life. It plays a positive role in enriching daily life, improving the living environment, shaping the urban image, and maintaining urban sustainable development [3,4]. Therefore, the lack or unbalanced allocation of resources itself is a kind of spatial injustice [5]. With the rapid development and continuous expansion of cities, the pressure on urban environments is gradually increasing. The uneven distribution and quality of UERSs lead to different degrees of imbalance between space supply and demand, which is difficult to maintain while meeting the actual needs of different groups [6]. Therefore, quantitative research on the supply and demand characteristics and the supply–demand matching relationship of UERS help to better understand the existing supply and demand situation of UERS and have important theoretical and practical values for improving the quality of UERS, the urban environment, and residents’ well-being.

**Figure 1:**
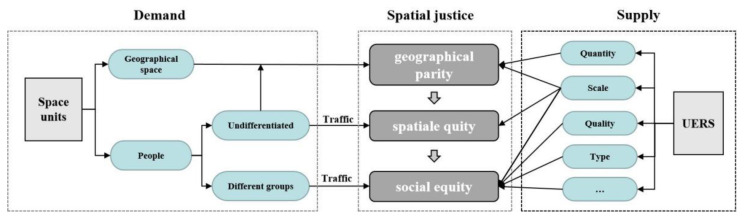
Evolution diagram of spatial justice theory.

In the process of urban regional development, the essence of spatial justice is to deal with the distribution of specific benefit of different groups in the process of resource allocation, and research on the supply–demand relationship of UERS is a typical problem of spatial justice research. The spatial justice is not absolute equity in the ideal sense but a kind of equity under the dynamic social relationship with historical development, which can be divided into three main stages: geographical parity, spatial equity, and social equity (Figure 1). The first stage of “geographical parity” is to solve the problem of the availability of UERSs in the region and to ensure that the amount of resource services per capita in different spatial units is basically equal [7,8,9]. The second stage of “spatial equity” not only pays attention to equal distribution of the UERS but also begins to emphasize the rationality of spatial layout of it. Therefore, the basic needs of people are paid attention, and scholars begin to explore the relationship between the allocation area of UERS and its corresponding service benefits [5,10,11]. In large cities with better development, a large-scale renovation is completed gradually and the development space inside the city is seriously limited. Cities gradually enter this stage of fine governance, which makes scholars and planners gradually pay attention to people’s democratic rights. Since then, the research on spatial justice has entered the third stage of “social equity”. This stage emphasizes that the abilities and needs of various groups are different, and it is necessary to ensure equal access to public service resources in different spaces and different socioeconomic groups and to advocate that services should be inclined toward specific vulnerable groups [12,13,14]. The case area of this study is Wuhan, a large city with a high level of development, so we study its supply–demand relationship in depth based on the social equity dimension.

The essence of the research on the supply–demand relationship of UERS is to analyze and establish the spatiotemporal relationship between supply and demand based on the quantitative evaluation of the supply of leisure and recreation services provided by UERS and the demand of urban residents for the service [15,16,17]. At present, scholars have carried out a lot of evaluation work on supply service capacity and value from the perspectives of social performance, ecological environment, and functional characteristics of URES [18,19,20]. In addition, with the increasing sensitivity of the interdependence between the natural environment and the living environment, the interaction between UERSs and residents is increasing. With “people” as the theme, the research on ecological recreation space such as the behavior, perception, and health characteristics of recreation people is also increasing, and the relationship between the supply and demand of ecological recreation resources is gradually being explored [21,22,23]. However, the existing supply–demand relationship of UERSs is mostly analyzed based on semi-quantitative methods such as a questionnaire method and an expert scoring method, which is highly subjective [24,25]. The research on the supply–demand relationship of UERS based on quantitative methods usually quantifies the spatial service capacity and residents’ demand through land use and population data, and the supply–demand relationship is measured by correlation analysis or accessibility calculation [26,27]. In existing studies, the supply level of UERS is expressed by scale indicators such as area and quantity, but the relevant factors such as ecological environment quality and the perfection of basic service facilities that directly affect the service ability of UERS and residents’ use experience are rarely reflected in the evaluation of the supply level [28,29,30].

The demand for UERSs is usually expressed by population density or population number, but people are generally regarded as undifferentiated individuals without considering the differences in abilities and needs of different groups such as gender and age. It results in the existing layout of UERS being still unable to meet the needs of residents, especially in vulnerable groups such as women, teenagers, and people who are older, whose needs and benefits are often neglected. For example, with the progress of society and the rise of feminism, the social status of women is constantly improving and their time available to participate in leisure activities is gradually increasing. However, women’s leisure time is still scattered because of their family roles, so they usually prefer UERSs with beautiful scenery and open spaces, which is different from the planning concept of “winding paths and secluded” in traditional gardens [31,32]. Furthermore, the “male standard” caused by the strong position of men in society has always been the mainstream of social life, and most existing urban public spaces are designed based on “de-genderization” or the “male standard”. To a certain extent, it ignores female characteristics and rights [33,34]. As teenagers are in a critical period of physical and mental growth, a good urban ecological environment and reasonable outdoor activities are vital to their physiological and psychological development [35]. In addition, the aging of large cities has been deepening in recent years, and the number of older people has been increasing. As the group with the strongest demand for UERSs, people who are older are more sensitive to the health and comfort of its use and have relatively high requirements for spatial functions and infrastructure, which are often ignored in the design of UERSs [36,37]. Therefore, this article selects females, teenagers, and people who are older as research subjects to explore the degree of social equity in UERS. In addition, census data are mostly used for resident data, with lagging time scale and low spatial accuracy [38]. However, the current urban demand characteristics are difficult to reflect in the results.

In view of this, taking the urban development area of Wuhan as an example, this paper selects indicators from the four aspects of basic environment, ecological environment, facility environment, and recreation satisfaction to evaluate the service quality of urban ecological recreation space and measures the supply service level enjoyed by residents based on community units. Combined with the demand characteristics of different groups, we analyze the supply–demand matching pattern of ecological recreation space in the study area and try to explore the shortcomings of the layout of ecological recreation space in the city to help urban ecosystem planning and livable city construction.

## 2. Materials and Methods

### 2.1. The Study Area

**Figure 2:**
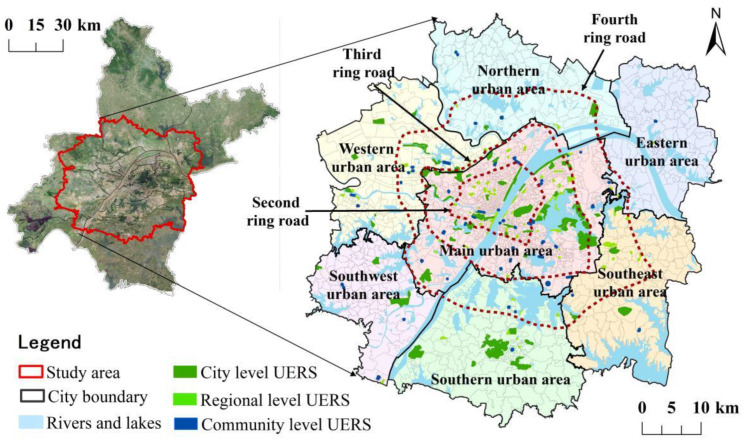
Map of the study area.

Although the overall greening level of Wuhan is among the best in the big cities, the phenomenon of “virtual high” of green space index in the built-up area with concentrated population is obvious. The green space difficult to use in daily life such as protected green space and green belt are included in the per capita green space area, which to some extent masks the problem of insufficient supply of ecological space for residents’ recreational activities in the city. Additionally, the service level of UERS in different regions is uneven, and the space quality is greatly different, which makes the use efficiency of each UERS very different. Therefore, we took the urban development area of Wuhan with concentrated residents as the research area. According to “the Master Plan of Wuhan 2010–2020” and “the Implementation Plan of “1 + 6” Spatial Development Strategy of Urban Development Area in Wuhan”, the township administrative boundary near the outer ring expressway is the basic boundary. It reaches Yangluo, Shuangliu, Zuoling, and Bao Xie in the east; Zoumaling, Caidian Chengguan Town, and Changfu in the west; Tianhe, Hengdian, and Sanli in the north; and Shamao, Jinkou, Zhengdian, and Wulijie in the south, involving 13 districts of Wuhan. Taking the community as the research unit, the boundary was locally adjusted. Finally, 1943 communities covered by the urban development area were selected as the research object (Figure 2), which covers the whole urban built-up area, with a total area of 3261 km^2^ and a total population of 8.3729 million in 2018.

### 2.2. Basic data and Processing

#### 2.2.1. Data of the UERS

UERS refers to all areas or places within the urban and suburban areas that have ecosystem service functions and can meet the daily outdoor recreation needs of residents, specifically including formal or informal space units with ecological properties and public service properties composed of natural or artificial landscapes, such as urban parks, botanical gardens, forest parks, wetland parks, leisure plazas, country parks, waterfront spaces, etc. [39].

According to the “Classification Standard of Urban Green Space” and “Urban Green Space System Planning of Wuhan city (2003–2020)”, we selected 213 spatial units with recreational functions in the urban development area as the research objects and divided them into three levels according to the spatial scale and visibility: city level, regional level, and community level (Figure 2 and Table 1). The city-level ecological recreation spaces mainly includes high-profile leisure and recreation places, tourist attractions, or large urban parks and waterfront spaces. It is the first choice for residents to travel on weekends and holidays, such as Hankou River Beach, Jiefang Park, Yellow Crane Tower Park, Moshan Resort, etc. The regional-level ecological recreation spaces mainly provide places for daily or weekend outdoor leisure activities for residents within the urban area, including small and medium-sized urban parks; sports parks; cultural sites; and large squares, such as Northwest Lake Square, Simeitang Park, Chuwangtai Ruins Park, etc. The community-level ecological recreation spaces are important places for daily entertainment, fitness, leisure, and communication activities of residents in the community, mainly including walk parks, street parks, and small squares near the residential area, such as Caidian Square, Baibuting garden, etc. Different levels of ecological recreation space can provide corresponding services and can radiate different recreation circles to meet the recreation needs of residents in different periods and ranges such as daily, weekends, and holidays. Using ArcGIS mapping software, we vectorize the UERS on a Gaode Map and satellite image map using the method of coordinate point positioning and visual interpretation, the point and polygon vector data of UERS are obtained, and the service range of each space is made by using the buffer method.

In addition, we also collected land use data, remote sensing image data, tourist comment data, facility data, and the survey data of UERS. The land use data were obtained from the GlobeLand30 produced by National Basic Geographic Information Center. (http://www.globallandcover.com/, accessed on 8 April 2021); the remote sensing image were obtained from the National Catalogue Service for Geographic Information (https://www.webmap.cn/, accessed on 10 April 2021); the tourist comment data were obtained from a social media website Dianping (https://www.dianping.com/, accessed on 4 June 2021); the facility data were obtained from Gaode Map (https://www.amap.com/, accessed on 14 April 2021); and the survey data were obtained through field survey in Wuhan UERS from 20 to 30 July 2021.

#### 2.2.2. Data of the Population

The residents’ population data is based on the 100 m resolution gridded population datasets provided by the official website of the WorldPOP project (https://www.worldpop.org/, accessed on 22 April 2021). Based on census data, supplemented by data such as remote sensing of night lighting, land use, distance factors to various land use types, and the information of elevation and slope, this dataset is acquired using random forest models and zonal densities method. It not only portrays the overall characteristics of all residents but also divides the population by gender and age groups and creates gridded datasets of different groups [40]. We use mask extraction methods to obtain population grid data for the study area and counts based on the administrative boundaries of the community units to obtain the number of residents in each community.

#### 2.2.3. Road Traffic Data

The urban road network is the link between residents and UERS and is the channel for residents to reach the UERS [41]. In this paper, the road information of the study area is extracted from remote sensing images and introduced into the ArcGIS platform to build an urban traffic network model. Additionally, according to the travel capacity of people walking and taking bus and subway, we calculate the shortest time for residents to reach each UERS through a network analysis.

### 2.3. Methods

#### 2.3.1. Quality Evaluation of UERS

**Figure 3:**
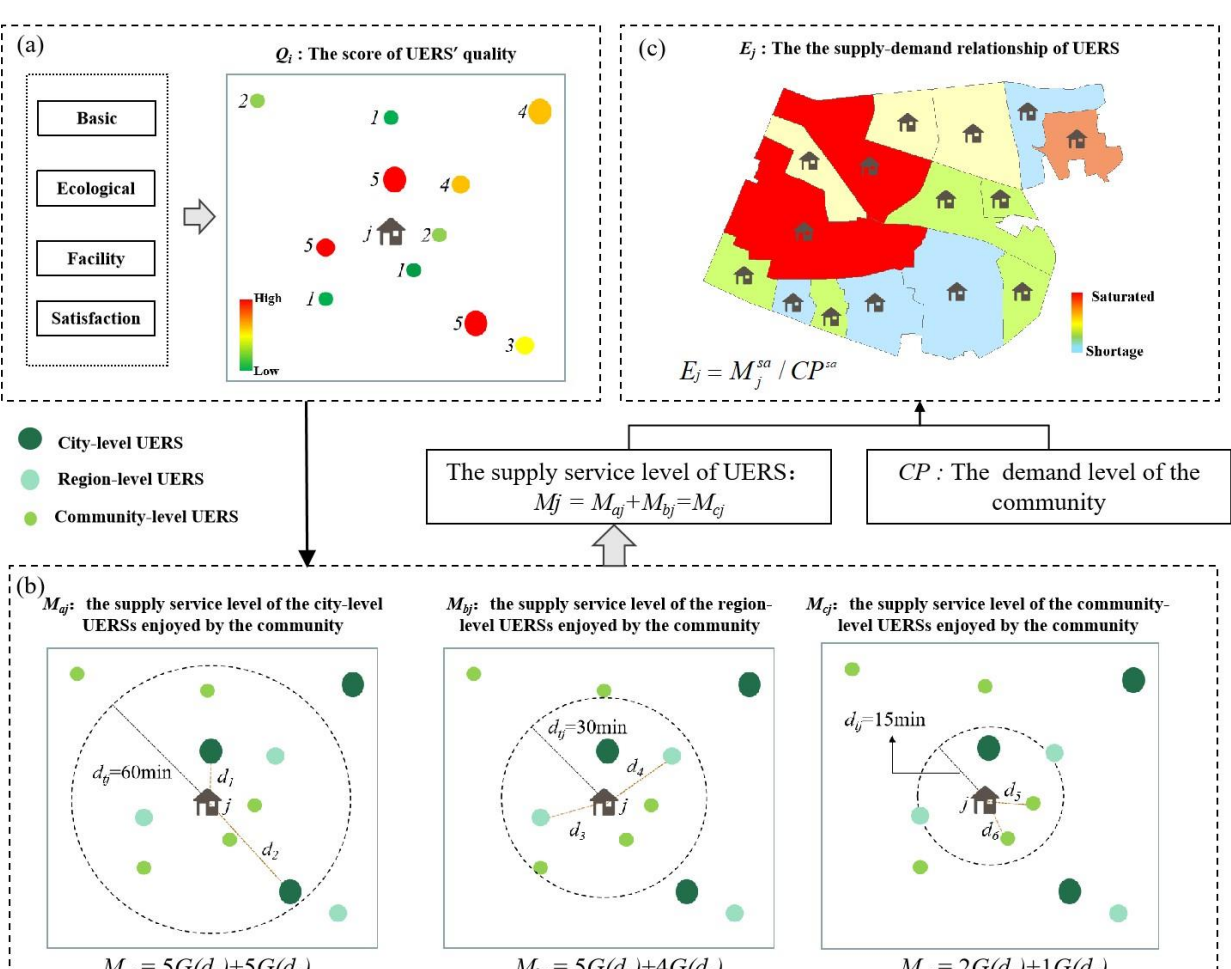
Quantification of the quality of UERS (**a**) and the supply level of UERS (**b**), and quantification of the supply–demand matching relationship of UERS (**c**).

The quality of UERS is a specific reflection of its service capacity and potential attraction. Considering the characteristic of the UERS and the target of this research, we selected the indicators as the quality evaluation elements of UERS from four dimensions: the basic environment, ecological environment, facility environment, and residents’ satisfaction. The indicators and detailed description are shown in Table 2. On this basis, the specific score of UERS’ quality level *Q_i_* is calculated using the weighting entropy method (Figure 3a) [42].

(1) $$P_{ig}=\frac{x_{ig}}{\sum_{i=1}^{n}x_{ig}}$$

(2) $$Hg=-k\sum_{i=1}^{n}P_{ig}\ln P_{ig},\;k=\frac{1}{\ln n}$$

where *i* denotes the UERS, *i* = 1, 2, …, *n*; *g* represents the quality index of UERS, *g* = 1, 2, …, *m*; *x_ig_* is the standardized value of the quality indicators; *P_ig_* is the ratio of the index value of UERS *i* to the total; and *H_g_* is the entropy of the quality indicators.

(3) $$w_{g}=\frac{1-H_{g}}{n-\sum_{g=1}^{m}H_{g}}$$

where *w_g_* denotes the weight of the quality indicators.

(4) $$Q_{i}=1000*\sum_{g=1}^{m}P_{ig}w_{g}$$

where *Q_i_* represent the quality level of UERS *i.* To further understand the quality level of UERS, the score is ranked from high to low and the quality level of UERS is divided into five grades by using the natural fracture method.

#### 2.3.2. Evaluation of Supply and Demand Levels of UERS

The supply service level reflects the quantity and quality of URES resources that can be obtained by different regions under the same conditions and can identify the insufficient areas of UERS, which is expressed by the total score of the quality of UERSs that each community can reach within the extreme travel time (*d*_0_). Due to the difference in the maximum travel time acceptable for residents to reach different levels of UERS, based on interviews and relevant research [43,44], we finally determine that the extreme travel times to reach city-level, regional-level, and community-level UERSs are 60 min, 30 min, and 15 min, respectively. Simultaneously, considering the differences in the distance between communities and different UERS, the distance attenuation coefficient (*G*(*d_tj_*)) is added to the formula to reflect that the service level of UERS enjoyed by community residents decreases with the increase in distance (Figure 3b).

(5) $$M_{tj}=\sum_{d_{tj}\leq d_{0}}^{N_{t}}Q_{t}G(d_{tj})$$

(6) $$M_{j}=M_{aj}+M_{bj}+M_{cj}$$

where *d_tj_* denotes the travel cost from the ecological recreation spaces to the community, which is expressed by travel time in this paper; *M_tj_* represents the supply level of UERS at the same level; *M_aj_*, *M_bj_*, and *M_cj_*, respectively, represent the supply service level of the city-level, region-level, and community-level UERSs enjoyed by the community; and *M_j_* refers to the overall supply service level enjoyed by the community.

The residents’ demand level (*CP*) reflects the potential demand of residents in different regions for UERS, expressed by the number of residents in each community unit.

#### 2.3.3. Measurement of the Supply–Demand Relationship of UERS

Based on the results of supply and demand levels enjoyed by different communities, the supply–demand index *E_j_* of each community is calculated using the following formula to analyze the balance between supply and demand for UERS (Figure 3c):

(7) $$E_{j}=M_{j}^{sa}/CP^{sa}$$

where $M_{j}^{sa}$ and *CP^sa^* represent the supply and demand levels after standardization. *E_j_* denotes the supply–demand matching index of each community, *E_j_* = 1 means that the supply and demand are relatively balanced, *E_j_* > 1 means oversupply, and *E_j_* < 1 means that the supply is less than the demand. In order to establish contact between the results of the supply–demand relationship and spatial justice, the calculated results of the supply–demand index are divided into six grades. The corresponding supply–demand index and the fairness degree of each situation are listed in Table 3.

## 3. Results

### 3.1. Analysis on the Quality Level of UERS

**Figure 4:**
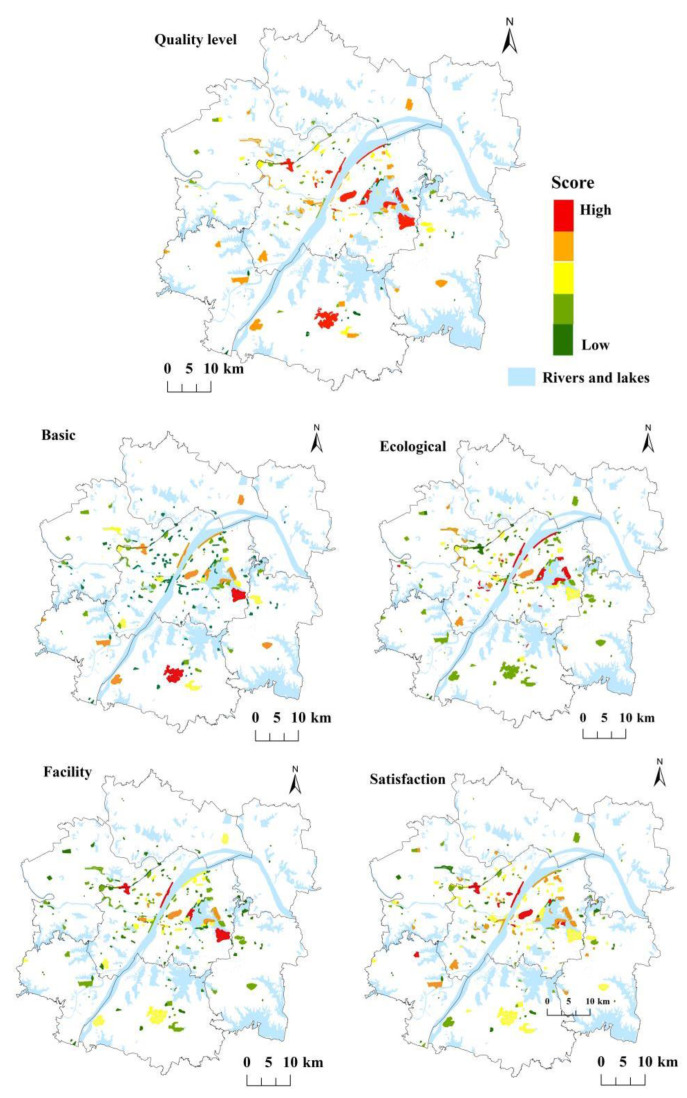
Spatial difference in the quality level of UERS.

Figure 4 reflects the spatial distribution characteristics of the quality level and corresponding indicators of UERS, and Table 4 makes statistics on the quality of UERS in different urban areas. Specifically, there are obvious differences in the distribution and quality level of ecological recreation space in different urban areas: both the quantity and quality of the UERS in the main urban area are much higher than those in the surrounding new urban areas. Although the scale of most UERS in the main urban area is relatively small, its ecological environment is good, the infrastructure service facilities are perfect, the landscape is more and distinctive, and the traffic environment is convenient. Therefore, the overall satisfaction of residents is relatively high. First-class UERSs with the highest quality in the main urban area are still large-scale and well-known scenic spots, such as East Lake, Wuhan Garden Expo Park, Hankou River Beach, etc. The UERSs of the surrounding new urban areas are mainly distributed in the six ecological wedges of Wuhan, showing the distribution characteristics of more in the southwest and less in the northeast. Among them, the number of URESs in the Western urban area is the largest. Except for the better ecological environment of some large-scale spaces, the scores of various indicators of other spaces are low, so the overall quality level is general. The UERS in the southern new urban area and the southwest new urban area is relatively good in average quality due to its large scale, some of which belong to scenic spots, which have a certain popularity and relatively high evaluation by residents. There are less than 10 ecological recreation spaces in the eastern and northern new towns, the quality levels are of the fourth and fifth levels, and the scores of various indicators are relatively low.

In summary, the quality of UERS is unevenly distributed in the urban development area of Wuhan based on all four dimensions. It can be seen that the first-class and second-class ecological recreation spaces with high-quality scores in the urban development area of Wuhan are mainly distributed in the main urban area and new towns in the southwest and south, showing the characteristics of distribution near the lake and along the river. As the landscape of rivers and lakes not only increases the ornamental value of UERS but also improves its ecological function to a certain extent, the ecological environment and recreation satisfaction of UERS along rivers and lakes are relatively high. The UERS with a low-quality score has a small scale and insufficient facilities and landscape, which is scattered in various urban areas.

### 3.2. Supply and Demand Characteristics of UERS

#### 3.2.1. Characteristics of the Supply Level

**Figure 5:**
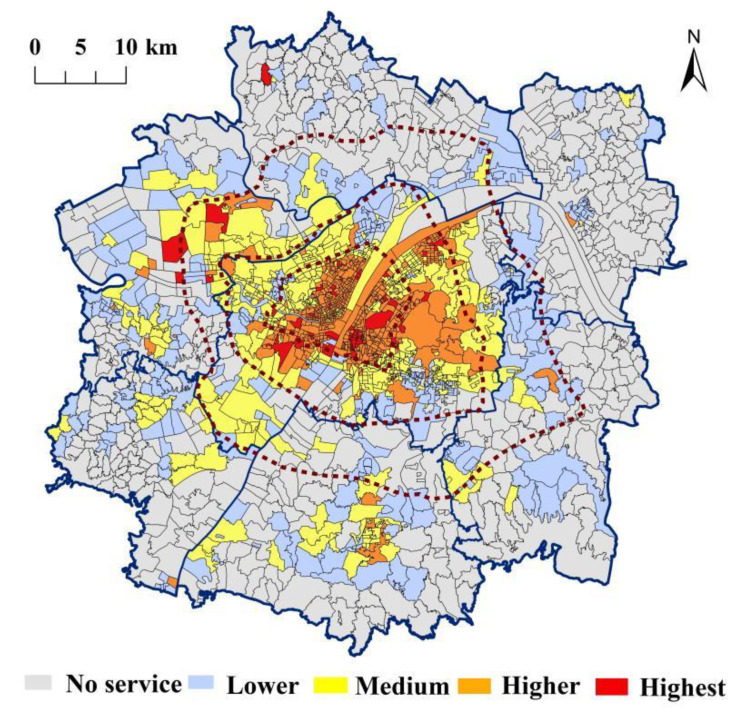
Spatial distribution map of the supply service level enjoyed by community residents.

Based on the service level calculation results of residents enjoying ecological recreation space, it is divided into five levels—high, relatively high, medium, low, and no service—and it is statistically and visually displayed by urban area (Table 5 and Figure 5). It can be seen that there are great differences in the supply service level of UERS enjoyed by residents in various communities in the urban development area of Wuhan. On the whole, the spatial distribution characteristics of UERS show a circular decreasing trend from the urban center to the periphery and shows the characteristics of the higher service level in the southwest and the lower service level in the northeast.

Specifically, there are few communities with a high service levels, more than 90% of which are distributed in the main urban area, of which Wuchang district is the largest, mainly because there are a large number of ecological recreation spaces in the core area of the main urban area and there are many high-quality ecological recreation spaces such as Hankou River Beach, Shahu Park, Yellow Crane Tower, Zhongshan Park, etc. Similarly, the communities with relatively high service level are concentrated within the Third Ring Road of the main urban area and near the Lingkonggang Development Zone in the southwest urban area, which is characterized by overall concentration and local dispersion. The reason for this is that the UERS in the main urban area is concentrated within the Third Ring Road and the UERS in the southwest new urban area is mainly distributed to the east of the urban area, close to the main urban area, so the overall supply service level of the communities in this area is relatively good. In addition, the communities with a general service level are mainly distributed at the edge of the main urban area and the areas with concentrated ecological recreation spaces in the surrounding new urban areas. In contrast, due to the uneven distribution of the UERS in the Wuhan urban development area and the small number of peripheral ecological recreation spaces, the communities with lower service levels and no services are mainly distributed in the periphery of the urban development area. The number of unserved communities in the six new towns accounts for more than 91%, and there were more than 100 unserved communities in the eastern and northern new towns.

#### 3.2.2. Characteristics of Demand Level

**Figure 6:**
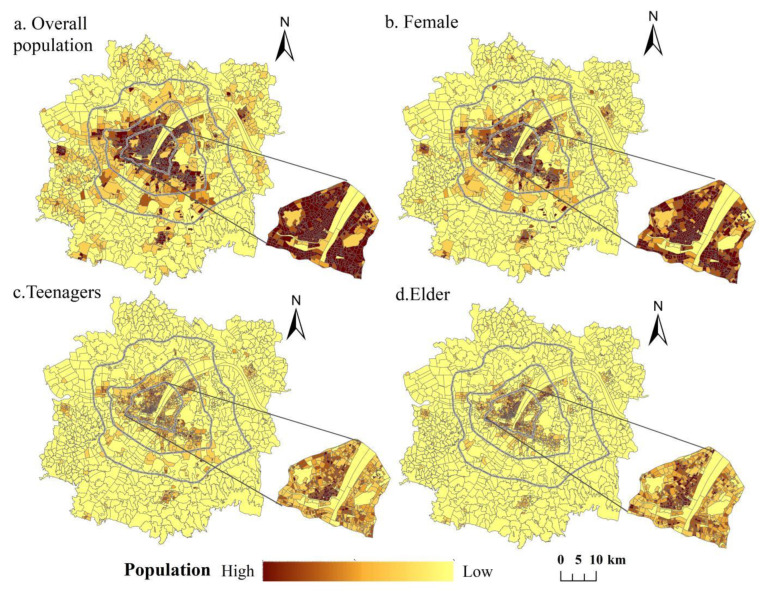
Spatial distribution of the residents’ supply of different groups.

Social spatial justice emphasizes the need to guarantee the equality of all residents’ enjoyment of spatial rights and interests, focuses on the differences in the abilities and needs of social groups, and advocates that public services should be inclined toward vulnerable groups. Therefore, we analyzed the demand characteristics of all residents and three special groups: female, teenagers, and people who are older in the urban development area of Wuhan (Figure 6).

As can be seen from Figure 6a, the spatial distribution of all residents’ demand is characterized by decreasing radiation from the center of the main urban area to the outside. Among the main urban areas, the three old urban areas of Jianghan District, Qiaokou District, and Wuchang District developed earlier and belong to the core living area of the city. There are many and concentrated residential communities, so the population density in this area is the highest and the demand for residents is the largest. Hanyang District and Jiang’an District take the second place, but there is a small difference from the above three urban areas. In contrast, Hongshan District developed later than the other central urban areas, but due to the existence of many universities and high-tech development zones, the developmental opportunities have attracted a large number of people, so the population density in this area is also relatively high. Qingshan District has the lowest population density among several central urban areas, and the demands of residents in each community are relatively fewer, but it is also much higher than that of the new surrounding urban areas. In the new surrounding urban areas, there are many industrial parks and dozens of small and medium-sized enterprises in the area near Wujiashan street in Dongxihu District of the western new urban area, and there are many residential areas to support their demands, so the population density and residents’ demand level are relatively high. Moreover, Zhifang Street in the southern new urban area is the core street of Jiangxia District, with rapid economic development under the radiation of the Optical Valley high-tech development zone and convenient transportation with the opening of Metro Line 7, so the population gathering area is well-formed here, and the demand level of residents is relatively high.

The spatial distribution characteristics of demand levels of different groups are basically consistent with all residents. The main urban area is relatively high and the surrounding new urban areas are relatively low, but there are still some differences in the local distribution (Figure 6b–d). The total number of females in the study area is about 4.0509 million, accounting for about 48% of the total. The high-demand areas are mainly concentrated within the Second Ring Road of the main urban area, and several high-demand cores are formed in Zhongnan Road Street, Huanghelou Street, Paper Square Street, the vicinity of Wansong Garden, and both banks of the Hanjiang River. The population of teenagers aged 0–18 in the study area is about 1.2906 million, accounting for about 15%, and the population of the elderly aged over 60 is about 0.9034 million, accounting for nearly 11%, which is a serious degree of aging [15,45]. Although the overall distribution characteristics of the teenagers and elderly groups are similar, the local characteristics still have subtle differences. First, the teenager groups are active and lively, with a larger range of activities, so the areas with high demand are slightly larger than that of people who are older; second, the teenager groups is at the stage of studying and pays more attention to educational resources, so the community youth population density in several streets with primary and secondary schools is large and the demand level is high. In contrast, most people who are older have retired, their physical conditions are not as good as that of the young groups, and their range of activities is limited, so the distribution of older groups is more concentrated in the main urban area with convenient transportation and perfect medical and living facilities.

### 3.3. Analysis on the Relationship between Supply and Demand of UERS

#### 3.3.1. Overall Supply–Demand Relationship of UERS

**Figure 7:**
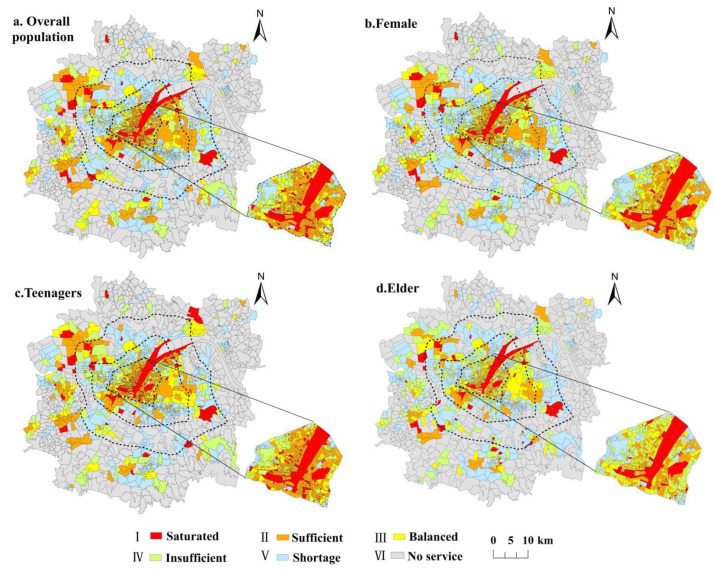
Supply–demand matching relationship of UERS in the urban development area of Wuhan.

**Figure 8:**
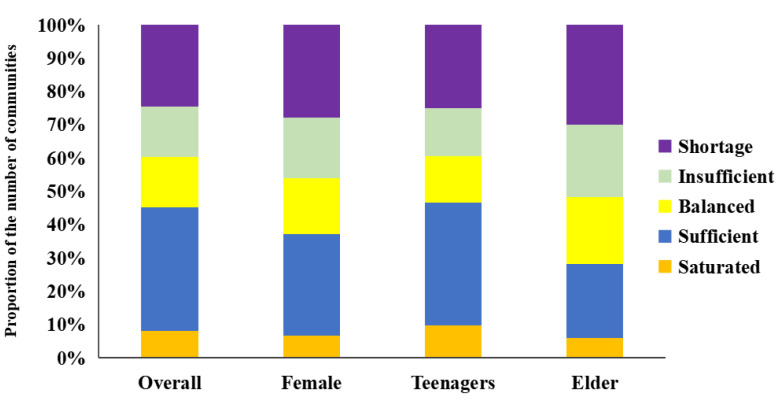
Proportion of communities of different groups at different supply and demand levels.

In terms of the matching characteristics of supply and demand of all residents (Figure 7a and Figure 8), there are only a few communities that can meet the demand of residents. which forms three agglomerations in the main urban area, the western urban area, and the southwest urban area, and other communities are scattered in the core streets of other urban areas. The supply deficit areas are distributed in the transition areas between the main urban area and new urban areas and near the core streets of the new urban areas. Due to accessibility issues, most communities in new urban areas are in a state of no service.

Specifically, among the communities services, the areas with saturated and sufficient supply are mainly located in some communities along the river in the main urban area and the communities with or close to large-scale UERS in the new urban areas, such as the Meizishan community, Wangjiaxiang, Xinhe community, etc. This oversupply phenomenon is mainly due to the presence or proximity of high-quality ecological recreation spaces in the community and the relatively low population density of residents within the community compared with surrounding communities. The balanced supply and demand communities are located in the vicinity of saturated supply and sufficient supply communities. Due to the radiation of these two types of regional ecological recreation space resource services, although the community population is relatively large, the supply and demand are at a medium level. The areas with insufficient supply in the main urban area are mainly located in Jiang’an District, Qingshan District, and Hongshan Optical Valley area. Compared with the new city, these were included in the built-up area earlier. Although the transportation is convenient, the population in the area is dense and the number of UERSs in the area is low, which is difficult to meet the daily activities of the residents. As the development of the new urban areas lags behind that of the main urban area, the population is relatively small and mainly concentrated in the core streets. Towards the periphery of the city, the number of URES becomes fewer, the density of the road network becomes less, and the accessibility become poor. Therefore, the areas without service and lack of supply are centrally connected and distributed in a ring around the periphery of the urban development area.

#### 3.3.2. Supply–Demand Relationship of UERS Represented by Different Groups

For the female groups (Figure 7b and Figure 8), the number of communities in each supply–demand matching level is at a medium level. Among them, the number of communities that can meet the demands of women (levels I–III) is lower than that of the whole and the teenagers, while the number of communities with supply deficit (levels IV and V) is higher than that of the whole. In the main urban areas where the female population is concentrated, most communities are in the situation of insufficient supply, except for the communities along the rivers and near the lakes and the communities with UERS. It indicates that, although there are convenient transportation and many UERSs in the main urban area, it is difficult to meet the actual needs of women because the female population is relatively concentrated and the quality of UERS is quite different. Therefore, when optimizing the UERS in the core area of the main city, we should pay more attention to the needs of women and the improvement of space quality.

For the teenager groups (Figure 7c and Figure 8), the hierarchical quantitative structure of supply and demand is similar to that of the whole, and the communities (Level I–Ⅱ) where supply exceeds the needs of teenagers are much higher than those of females and the elderly. The communities are mainly concentrated within the Second Ring Road of the main urban area, in the Linkonggang area of the western urban area, Daji and Zhuankou streets of the southwestern urban area. However, the number of communities with balanced supply and demand is few, indicating that the fairness of their spatial distribution is poor. Similarly, the proportion of communities with insufficient supply and lack of supply (level IV-V) is significantly lower than that of females and people who are older but still accounts for a large proportion, and these communities are mainly distributed in new cities, indicating that the supply of UERS in new urban areas still cannot meet the needs of teenagers and so needs to be improved.

For the older groups (Figure 7d and Figure 8), the quantitative structural characteristics of the supply–demand relationship levels are significantly different from other groups. Compared with other groups, although the number of communities with balanced supply and demand is the largest, the number of communities at levels IV and V is highest and the number of communities at levels I and II is lowest. This indicates that, although the fairness among the older groups is the best, the needs of people who are older in most communities have not been met. The demand for the older population in the main urban area is relatively large and concentrated. Although the overall supply in the core area of the main urban area is sufficient, the spatial distribution is uneven, resulting in an obvious accumulation area of insufficient supply in Qingshan District and Qiaokou District. In contrast, in new urban areas, the older population is smaller and most communities with UERS are in ‘saturated’ or ‘sufficient’ supply. However, people who are older are affected by their physical condition and have a limited scope of activities. They usually choose spaces close to their home for recreation, so most communities without UERS are ‘insufficient’ or ‘shortage’ supplied, and some of those were even no-service communities.

To sum up, there are obvious differences in the supply–demand matching relationship of UERS among diversified social groups, but the overall equity level is low and the supply and demand of UERS in most communities are in a mismatched state. Among them, except for communities without services, teenagers are in an unbalanced state of supply surplus, while women and people who are older are in an unbalanced state of supply deficit.

## 4. Discussion

### 4.1. Quality Evaluation of UERS Based on Multiple Dimensions

The supply–demand relationship of UERS is essentially a man–land relationship, that is, the supply–demand relationship between people in space and scarce ecological recreation resources. Its local characteristics can reflect the overall development of the city to a certain extent [46]. At present, research on the relationship between supply and demand of UERSs is usually only concerned with the quantitative surplus and deficit between supply and demand, and deeper information mining is still insufficient [47]. Starting from the quality dimension of UERS and considering its basic environment, ecological environment, facility landscape, and recreation satisfaction, we constructed a relatively complete UERS quality evaluation system to comprehensively measure the quality level and service ability of UERS. The proposed evaluation system more accurately measures the supply–demand matching relationship of UERS. Compared with existing research [48,49], the research results can better identify areas with insufficient supply of UERS in the main urban area and can effectively guide the optimal layout of UERS in the main urban area.

### 4.2. Selection of Differentiated Travel Threshold Based on the UERS Level

The travel psychological threshold of residents determines the potential activity range of residents and directly affects the supply level of residents’ access to UERSs. However, in the existing studies on the supply level of UERS, the impact of different transportation modes on the selection of thresholds is mostly considered, and the change in psychological threshold caused by the grade difference of ecological recreation space is rarely considered [50,51]. Through the investigation of Wuhan citizens, we found that there are differences in the limited travel times that residents accept to reach different types and levels of ecological recreation spaces. The higher the level of ecological recreation space, the longer the residents can tolerate for travel time to reach that space. Based on this, according to the psychological thresholds corresponding to different levels, we calculated the degree of community supplied at this level and emphasized the importance of the level of UERS to residents’ travel choices.

### 4.3. Considering the Differences in Population Characteristics

The theory of spatial justice emphasizes that the supply–demand equity of UERS should consider not only spatial equality but also the factors of social equity [52,53,54]. Different groups have different preferences for the UERS, such as the female group preferring natural scenes or a comprehensive UERS on a large scale, beautiful natural environment, and rich activities; teenagers being energetic, lively, and eager for knowledge and thus preferring spaces with rich sports facilities, or zoos and botanical gardens to facilitate knowledge exploration; and people who are older valuing physical exercise, communication, and making friends as their most important demands of a UERS. Therefore, a UERS close to home with a large activity space and rich facilities would be more popular. We added the demands of specific social groups to gain a deeper understanding of the spatial differences in supply–demand relationships for different groups. In addition, we based this paper on micro-scale community unit so that it is closer to the real living units of residents, which has an important reference value for urban fine management [55].

### 4.4. UERS Policy Suggestions

Based on the above analysis, we offer a few suggestions to inform about future urban planning and guide policy making based on inference from our findings: (1) The main urban area is rich in ecological recreation resources, and the quality of the URES should be improve. Due to the limited land resources in the main urban area, a new and large-scale UERS is less likely to be added. For areas with an insufficient supply of people who are older and of females, we should pay attention to improvements in spatial quality according to the specific needs of the different groups, such as adding entertainment and infrastructure suitable for people who are older and optimizing the ecological environment of UERS. At the same time, we should improve the accessibility of public transport in ecological recreation spaces and improve the convenience for residents to use the space and improve their feelings about using the space. (2) Small and medium-sized parks and green spaces should be added in the core streets of new surrounding urban areas as required, and multiple mini-community parks and squares should be built in unserved areas. The population in the new surrounding urban areas is concentrated in the core streets, and most communities in new urban areas are undersupplied or lacking in supply, so we should add small or medium-sized parks within the core streets to improve the supply–demand levels in these area. The edge areas of new urban areas are mostly no-service communities, so small pocket parks or small plazas should be built to meet the daily recreational needs of the residents. (3) Land resources and the advantages of an ecological background for new urban areas should be made use of to create a large-scale comprehensive UERS. There are six green wedges in the new surrounding towns, with excellent ecological background resources, but the recreational conversion rate of ecological green space is relatively low, so the quantity and quality of ecological recreational spaces are small. Therefore, we should make full use of the advantages of natural resources to plan and build a comprehensive ecological recreation space. At the same time, compared with people who are older, women and teenagers have a wider range of activities. A large-scale comprehensive ecological recreation space will further meet the recreation needs of these groups.

### 4.5. Limitations and Future Research

There are some limitations in this study. First, we selected walking as the only mode of transportation to access the UERS, but in fact, the modes of transportation used by residents is varied, such as bicycles, buses, subways, private cars, and a combination of transportation modes. Therefore, multiple modes of transportation will be considered in future studies of UERS. In addition, in the classification of UERS, we only divided the UERS into the city level, the regional level, and the community level according to the spatial scale and popularity and did not consider the residents’ demand of the function of UERS. Consequently, the classification of UERS based on its functional characteristics are suggested to be taken into account in future studies of the supply–demand relationship of the UERS to enhance the validity and relevance of policy suggestions.

## 5. Conclusions

Taking a Wuhan urban development area as an example, we evaluated the supply and demand levels of UERS and quantified the spatial relationship between residents and UERS. The results showed that 72% of communities in the Wuhan urban development area can obtain the services of UERS within the limited travel time, indicating that most residents can benefit from the services of the UERS. However, these communities are mainly distributed in a main urban area and the core streets of the new urban area, and there are still large areas of communities in the periphery of the city that remain unserved. Moreover, due to the uneven distribution and large difference in quality levels of the UERS, there is still a large disparity in the supply level enjoyed by communities with the service. The overall supply level enjoyed by these communities is higher in the main urban area and lower in the new urban area, which is characterized by a decreasing trend of radiation from the urban center to the periphery. In terms of the supply–demand matching relationship, only about 20% of the communities experience a balance in the supply and demand, and most communities experience an imbalance, indicating that the matching relationship between supply and demand in the Wuhan urban development area is not ideal and that there is a serious spatial mismatch in the UERS resources within the whole city. In addition, we found that there are existing spatial disparities of the supply–demand relationship of UERS among the multiple social groups in Wuhan. Therefore, the quantity of UERSs should not only increase to balance its spatial distribution but also improve the quality of UERS to adjust the equity of UERS enjoyed by different communities and different groups to realize coordinated development of the city.

## Figures and Tables

**Table 1:** Classification standard and basic information of urban ecological recreational space.

Space Level	Number	Area (hm^2^)	Decision Criteria	Service Radius (m)
City level	72	7412.17	*S_i_* ≥ 25 hm^2^	3000
Regional level	85	1278.66	10 hm^2^ ≤ *S_i_ <* 25 hm^2^	2000
Community level	56	153.87	0 hm^2^ ≤ *S_i_* < 10 hm^2^	1000

**Table 2:** UERS quality measurement indicators and data description.

Dimensions	Indicators	Description	Weight
**Basic**	Area (*X*_1_)	It reflects the scale of UERS.	0.072
	Shape index (*X*_2_)	It represents the complexity of the shape of UERS and is calculated by dividing the area by the perimeter.	0.089
	Hygiene (*X*_3_)	It reflects the quality of the sanitary environment of the UERS. The sanitary environment is scored as 0–10 points based on the network street view image, public comments in DianPing, and field research.	0.012
	Environmental carrying capacity (*X*_4_)	It reflects the number of people that can be accommodated in the ecological recreation space in theory. Referring to the code for the design of parks in 2020 and relevant studies, the higher the level of UERS, the greater the proportion of water and slope area in the space and the larger the per capita area. Finally, it is determined that the per capita area occupied area is 25 m^2^–80 m^2^ per person, and the environmental carrying capacity is calculated by dividing the space area by the per capita area.	0.077
**Ecological**	Water ratio (*X*_5_)	It is the ratio of water area to total space area, which is calculated based on land use data extraction.	0.184
	Vegetation coverage (*X*_6_)	It is the ratio of vegetation-covered area to total space area, which is calculated based on land use data.	0.045
	Temperature regulation (*X*_7_)	It reflects the impact of UERS on temperature and environment. Based on the remote sensing image data, the actual spatial surface temperature was calculated using ground temperature inversion methods, and the ability to reduce temperature was expressed as the difference between the actual spatial temperature and the average urban temperature.	0.020
**Facility**	External facilities (*X*_8_)	It is the number of public transport, catering facilities, parking lots, shopping facilities, and other supporting service facilities within the space service radius.	0.048
	Internal facilities (*X*_9_)	It is the number of recreational and sports facilities, toilets, parking lots, convenience stores, and other service facilities in the UERS.	0.081
	Landscape (*X*_10_)	It is the number of pavilions and landmark landscape in the UERS.	0.062
	Road length (*X*_11_)	It is the internal road length of UERS, which is vectorized based on network map and remote sensing image.	0.060
**Satisfaction**	Attention (*X*_12_)	It is the total number of public comments on the UERS.	0.053
	Favorable comment (*X*_13_)	It is the number of positive comments on the UERS.	0.053
	Score (*X*_14_)	It is the users’ rating of the UERS.	0.144

**Table 3:** Meaning of supply–demand matching and fairness.

Class	Supply–Demand Matching	Fairness	Value Range
I	Saturated	Serious inequity	5 < *E_j_*
II	Sufficient	More equity	1.25 < *E_j_* ≤ 5
III	Balanced	equity	0.75 < *E_j_* ≤ 1.25
IV	Insufficient	Inequity	0.35 < *E_j_* ≤ 0.75
V	Shortage	Serious inequity	0 < *E_j_* ≤ 0.35
VI	No service	Serious inequity	*E_j_* = 0

**Table 4:** Statistics on the quality level of ecological recreational space in different urban areas.

	Number	Area (hm^2^)	Basic	Ecological	Facility	Satisfaction	Quality Level	Average Quality Level
Main urban area	134	4539.93	154.62	181.59	192.89	186.39	715.49	5.34
Easter urban area	3	23.84	1.38	1.70	1.42	2.65	7.16	2.39
Western urban area	26	796.71	22.94	30.12	18.42	21.07	92.55	3.56
Southern urban area	17	1758.59	30.53	15.21	14.98	12.34	73.06	4.30
Northern urban area	8	279.72	10.91	3.72	4.97	6.17	25.77	3.22
Southeast urban area	15	677.54	14.64	12.42	9.34	12.54	48.95	3.26
Southwest urban area	10	490.00	14.97	5.23	7.98	8.85	37.03	3.70

**Table 5:** Statistics of residents’ supply service level in different urban areas.

	Highest	Higher	Medium	Lower	No Service
Main urban area	54	525	354	114	48
Easter urban area	0	1	2	19	116
Western urban area	4	8	55	60	51
Southern urban area	0	5	35	37	81
Northern urban area	1	0	3	29	103
Southeast urban area	0	1	9	36	71
Southwest urban area	0	1	14	32	74
Total	59	541	472	327	544

## Data Availability

Not applicable.
